# Mental health and psychosocial support for asylum seekers and refugees in Greece: a narrative review of stressors, needs, services, and barriers

**DOI:** 10.1186/s12939-026-02786-2

**Published:** 2026-02-25

**Authors:** Lars Dumke, Eleni Maousidi-Ziganou, Penny Kalpaxi, Ledia Lazeri, Jennifer Hall, Joao Breda

**Affiliations:** 1 WHO Athens Office on Quality of Care and Patient Safety, WHO Regional Office for Europe, Athens, Greece; 2 Department of Psychiatry and Psychotherapy, University Medical Center Hamburg-Eppendorf, Hamburg, Germany; 3 WHO Regional Office for Europe, Copenhagen, Denmark

**Keywords:** Mental health, Refugees, Access, Equity, Barriers, Europe, Review

## Abstract

**Background:**

Over the past decade, Greece has received a high number of applications for international protection, creating an urgent need for comprehensive mental health and psychosocial support (MHPSS) systems for asylum seekers and refugees. Despite its central role in the European migration context, there is currently no synthesis of evidence on MHPSS for these populations, which limits the understanding of inequities in mental health outcomes and access to care.

**Aims:**

This narrative review synthesizes the available evidence on major sources of distress, mental health needs, the landscape of MHPSS services, and barriers to access for asylum seekers and refugees in Greece.

**Method:**

We conducted a narrative review adhering to guidance from the Inter-Agency Standing Committee (IASC) on multi-sectoral MHPSS needs and resources assessments. Literature published in English or Greek between January 2015 and May 2025 was identified through PubMed, Web of Science, Google Scholar, and targeted web searches. Twenty-five journal articles and five additional sources (situation reports, assessments, and policy documents) were included.

**Results:**

The reviewed literature highlights the significant mental health needs of asylum seekers and refugees in Greece. According to the available studies, at least one third of asylum seekers and refugees experience mental health problems, such as depression or PTSD. These elevated mental health needs are linked to displacement, including post-migration stressors, as a critical social determinant of health. Despite considerable efforts to improve MHPSS, access to services and their quality are limited by structural barriers that disproportionately affect asylum seekers and refugees. These include a limited public mental health system, restrictive policies hindering inclusion into national systems, insufficient workforce capacity and inadequate adaptation of services to cultural and contextual needs.

**Conclusions:**

Asylum seekers and refugees in Greece experience inequities in both mental health outcomes and access to adequate care. Strengthening MHPSS for asylum seekers and refugees requires a multi-layered approach that addresses social determinants, integrates MHPSS for refugees into national systems, enhances community-based support, builds workforce capacity and competence, and improves monitoring and evaluation. Equity-focused recommendations are outlined to guide policy and practice.

**Clinical trial number:**

Not applicable.

Greece is one of the main entry points into Europe for migrants. Major drivers of migration include armed conflict and instability in the Middle East and parts of Africa. Many of those arriving in Greece have fled war, persecution, and human rights violations, often enduring severe trauma before and during their migration journeys [1–3].

In 2015 and 2016, Greece experienced an unprecedented increase in arrivals, with more than one million individuals reaching its borders [4]. While the majority continued their journey toward Northern and Western Europe, this dynamic began to shift in recent years, gradually transforming Greece into a host country for refugees and migrants [1]. In 2024, Greece recorded more than 70,000 new applications for international protection [5]. By mid-2024, Greece was hosting approximately 240,000 asylum seekers and refugees (see Fig. 1 for key characteristics) [6].

**Fig. 1:**
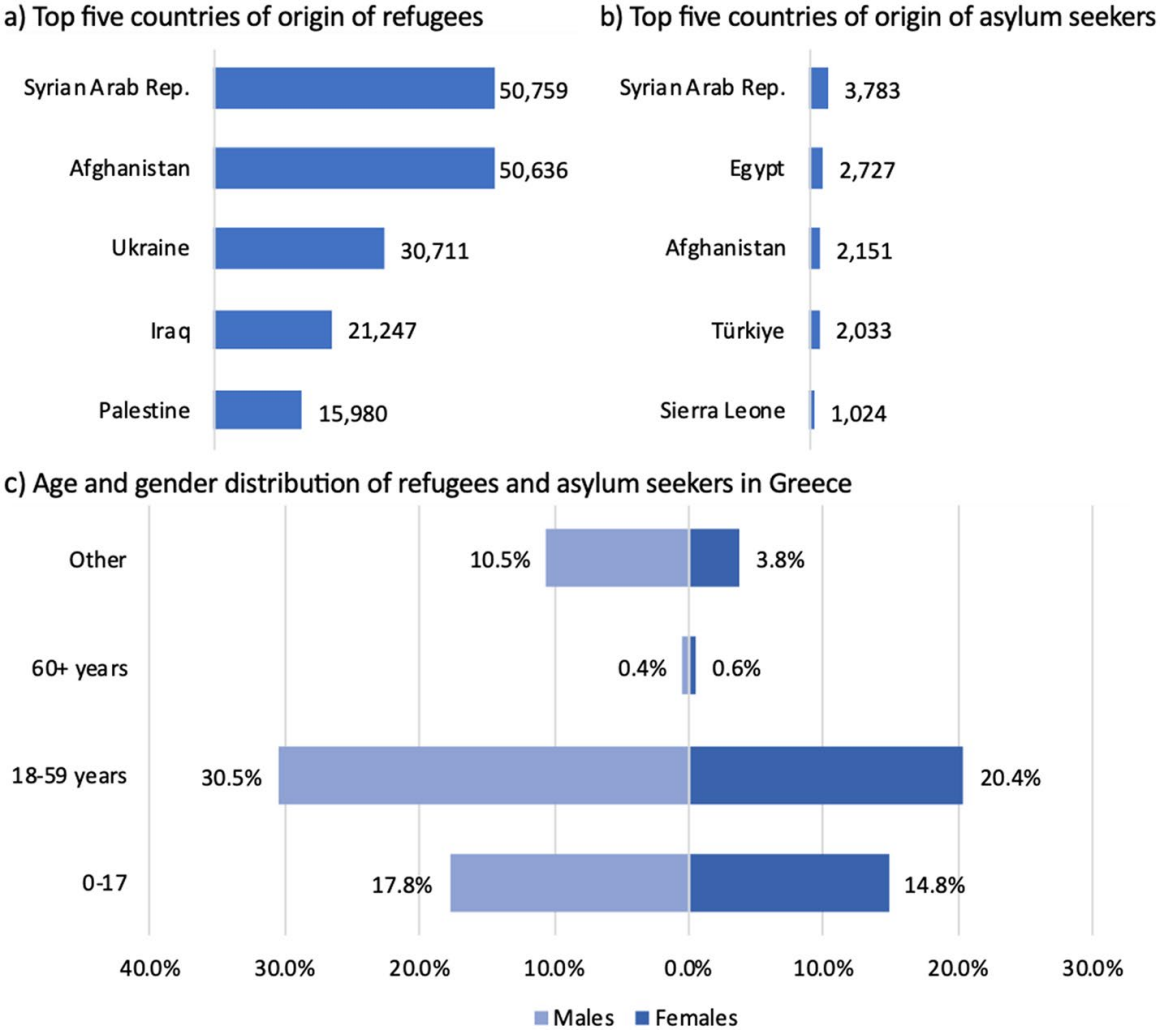
Key characteristics of asylum seekers and refugees in Greece. Note: Data derived from UNHCR Refugee Data Finder based on data available as of April 2025 [6]

With the increasing number of individuals seeking international protection and building their lives in Greece, the need for comprehensive support systems, including mental health and psychosocial support (MHPSS), has become more critical than ever. People seeking international protection are often exposed to multiple risk factors for poor mental health along the migration trajectory, including potentially traumatic experiences, loss, disruption of social networks and post-migration stressors. Systematic reviews indicate that approximately one third of refugees experience mental health disorders such as depression, post-traumatic stress disorder (PTSD), or anxiety, with prevalence rates substantially higher than those observed in the general population [7, 8]. Without adequate and timely support, these mental health problems may persist and have far-reaching consequences for individuals, families, and communities. However, access to appropriate mental health care is frequently limited for refugees and asylum seekers, including in European host countries [9, 10].

As emphasized by the World Health Organization (WHO), refugees and migrants are entitled to the same universal human rights and fundamental freedoms as other people, including mental health care [11]. Ensuring access to adequate MHPSS is not only a legal and ethical obligation but also a crucial factor in promoting successful integration into host communities [12]. While a substantial body of research has documented the prevalence of mental health problems and exposure to adversity among displaced populations globally, evidence remains fragmented with regard to country-specific contexts, particularly concerning the organization of MHPSS services, patterns of service availability, and context-specific barriers to care [9]. Yet, such insights are critical for guiding the design, implementation, and improvement of MHPSS systems that are responsive to the specific experiences and challenges of asylum seekers and refugees [12]. In Greece, despite its central role in European migration routes, a comprehensive synthesis of evidence on stressors, mental health needs, available MHPSS services, and barriers to care for asylum seekers and refugees has been lacking.

This narrative review examines the MHPSS needs and resources for asylum seekers (i.e., individuals who are seeking international protection) and refugees (i.e., individuals who have applied for asylum and have been granted international protection) in Greece. The review focuses specifically on asylum seekers and refugees, as these groups are subject to distinct legal frameworks, reception conditions, and policy arrangements that directly shape their access to mental health and psychosocial support services. It aims to provide an overview of key issues, including the identification of MHPSS needs, relevant contextual factors and determinants, the availability of services, and existing barriers and gaps. By synthesizing information from diverse academic and humanitarian sources, this review aims to contribute to the broader literature on refugee mental health and social inclusion. It aims to inform policymakers, humanitarian actors, and public health practitioners seeking to strengthen equitable MHPSS services for asylum seekers and refugees in Greece and beyond.

## Methods

A narrative review approach was employed to synthesize the available evidence on MHPSS for asylum seekers and refugees in Greece. This method was considered appropriate given that the review aimed to provide a comprehensive overview across a broad range of key topics in the field of MHPSS, as well as the anticipated heterogeneity of sources, which included academic research, situation reports, and policy documents. In health services research, narrative reviews enable the integration of diverse literature to identify research gaps and provide insights for advancing the field, especially in areas that remain under-researched or under-reported [13, 14]. Our review was guided by the Inter-Agency Standing Committee (IASC) *Multi-sectoral MHPSS Needs and Resources Assessments Toolkit*, which emphasizes narrative reviews as a means to consolidate existing evidence to inform equitable and efficient mental health support planning [15]. Mental health and psychosocial support in this review is understood in accordance with the IASC guidelines on MHPSS in emergency settings [16], which encompasses a spectrum of interventions, including basic services and security, community and family support, focused non-specialized supports, and specialized mental health care.

A comprehensive literature search was conducted between January and May 2025. Relevant literature was identified through the databases PubMed and Web of Science, and Google Scholar. In addition, targeted searches were conducted using Google Search as a search engine to identify relevant grey literature. Search terms combined keywords related to mental health and psychosocial support (e.g., “mental health”, “psychosocial”, “MHPSS”) with terms related to access, help-seeking, and service utilization (e.g., “needs”, “access to care”, “help-seeking”, “service utilization”), as well as population and context descriptors (“asylum seekers”, “refugees”, “Greece”). Additional materials were identified through manual screening of reference lists and websites of NGOs, international organisations, and government sources. We included a broad range of documents: peer-reviewed empirical studies (qualitative, quantitative, or mixed-methods), assessment reports and situation analyses from international organizations and NGOs, project evaluations, humanitarian needs assessments, and national or international policy documents relevant to MHPSS for refugees and asylum seekers. Documents were excluded if they did not focus on mental health or psychosocial support or did not address refugees, asylum seekers, or the Greek context. Sources published in English or Greek between January 2015 and May 2025 were considered to capture developments following the 2015–2016 increase in refugee arrivals to Greece. In total, 25 journal articles and five additional sources (situation reports, assessments, and policy documents) were included. Data on mental health needs were extracted using a structured template capturing publication type, population, setting, methodology, and key findings. A narrative synthesis approach was used to identify patterns related to key topics in MHPSS, guided by the *IASC MHPSS Needs and Resources Assessments Toolkit* [15], including relevant policies, available services, sources of distress, help-seeking behaviours, mental health service utilization and barriers to accessing care. To structure the identification and presentation of barriers, the Levesque patient-centred framework for access to health care was applied [17]. This framework helps to systematically capture the dynamic interaction between health system characteristics and individual capacities to seek, reach, engage, and receive appropriate care. A multidisciplinary research team, including mental health professionals, public health experts, and political scientists, conducted the screening, review and narrative synthesis. All team members are co-authors of this paper. The initial screening and identification of relevant literature were conducted independently by two authors (LD and EM). The in-depth review and synthesis were led by the first author and informed through iterative discussion and critical input from all co-authors, drawing on their respective clinical, research, and policy expertise.

## Results

### Current landscape of MHPSS services for asylum seekers and refugees in greece

Mental health services for asylum seekers and refugees in Greece are available through a mix of camp-based health units, public sector health institutions, and NGOs.

For asylum seekers, the primary point of contact for mental health and psychosocial support is the on-site health care and psychosocial support unit in the accommodation facilities. Since July 2024, these units have been managed under the IPPOKRATIS programme, led by the International Organisation for Migration (IOM) in cooperation with the Ministry of Migration and Asylum [18]. According to the programme, psychosocial support teams have been established to work alongside with medical teams. These medical and psychosocial support unit are intended to be staffed with specialized personnel, including social workers and psychologists, providing individualized psychosocial care. Referrals to secondary and tertiary health care structures are made when clinically necessary. However, based on the reviewed literature, psychosocial services were in the past not consistently available across all camps, and it remains unclear to what extent they are integrated into and connected with the public health system [19, 20].

Asylum seekers and refugees in Greece can use the public mental health system, including psychiatric hospitals, psychiatric departments in general hospitals, and community-based mental health services [21]. Access to these services is possible through the use of the Provisional Insurance and Health Care Number (PAAYPA) for those seeking international protection, or the Social Security Number (AMKA) for recognized refugees and beneficiaries of subsidiary protection. However, challenges in obtaining the required documentation persist and the percentage of refugees with AMKA has significantly decreased from 77% in 2023 to 68% in 2024 [22]. In the past years, access to public health care services has been reported to be constrained [23, 24], including for vulnerable population groups such as unaccompanied refugee minors [19, 25]. The Greek national health system, already under strain following the 2009 financial crisis, continues to face major capacity challenges and there is often a lack of available public mental health services, especially in remote areas where accommodation facilities are located [24, 25].

In this context, NGOs, church charities and local communities have often played a vital role in filling critical service gaps [26, 27]. NGO-operated MHPSS programs, including mobile mental health units and medical centres, were in the past particular important in addressing the needs of vulnerable groups and emergency cases [24, 28]. However, the availability of these services often depends on short-term funding cycles, which can limit continuity and long-term planning and cannot cover all needs [26].

Overall, from the materials reviewed, there is a lack of clarity which mental health services are available both within and outside the camps. Referral pathways are described as fragmented and public mental health services are under-resourced and inconsistently distributed, particularly in island and remote mainland locations [20, 23]. Overall, the availability and quality of MHPSS may vary significantly from one camp to another, depending on the staffing of camp-based health care and psychosocial support units, the proximity to public healthcare services, and the presence and extent of NGO-supported programs. Transitions from camp-based accommodation to urban settings or independent housing are often associated with the withdrawal of structured support mechanisms, resulting in increased exposure to stressors such as housing insecurity, loss of material assistance, food insecurity, and reduced access to essential services, including MHPSS [19, 29, 30]. Barriers to accessing services are discussed in more detail below.

### Major sources of distress among asylum seekers and refugees in Greece

Asylum seekers and refugees in Greece often experience multiple sources of distress, spanning experiences before, during, and after migration (see Table 1).

**Table 1:** Major sources of distress among asylum seekers and refugees in Greece

Common sources of distress
Pre- and peri-migration trauma • Exposure to war, violence, persecution • Life-threatening experiences during migration, particularly sea crossings • Gender-based violence
Experiences of loss and grief • Loss of family members through death, disappearance or forced separation • Loss of home, livelihood, social status • Disruption of family structures and support networks • Loss of identity and belonging
Stressors in the current living context • Overcrowded, unsafe camp conditions • Lack of privacy and autonomy • Discrimination and mistreatment • Prolonged asylum procedures, uncertainty, fear of deportation • Limited access to education, employment, and integration opportunities • Limited access to (mental) health services • Social isolation and marginalization

#### Pre- and peri-migration trauma

Exposure to conflict-related violence and life-threatening experiences before and during migration are frequently reported as major contributors to psychological distress [2, 26, 31, 32]. For example, female asylum seekers and refugees from Afghanistan highlighted experiences of gender-based violence, while men often reported experiences of armed conflict and persecution in their countries of origin [32]. Refugees also frequently identified the migration journey itself, including experiences during border crossings, as a significant source of psychological distress [33].

#### Experiences of loss and grief

Loss and grief represented central issues throughout the reviewed literature. Asylum seekers and refugees frequently report the loss of loved ones, whether through death, disappearance, or forced separation [2]. At the same time, it was reported that displacement also encompasses the loss of home, community, social status, livelihoods, and a sense of identity and belonging [2, 32]. The disruption of family structures and the absence of familiar social support networks were described as contributing to profound emotional distress [2, 32].

#### Stressors in the current living context

Several studies discuss challenges associated with daily life in Greece and living conditions in the camps [3, 33, 34]. Asylum seekers and refugees frequently described camps as overcrowded, poorly maintained, and lacking in privacy, legal, and health support [2, 32]. Among Syrian refugees in northern Greece, those residing in urban centers showed better physical and mental health compared to those living in other types of accommodation [30]. Qualitative studies highlight a sense of insecurity and threat among refugees and asylum seekers, due to experiences of violence within camps, mistreatment by officials, and discrimination [31, 33]. Prolonged asylum procedures, lack of clear information, and fear of deportation were identified as creating uncertainty, contributing to feelings of hopelessness, lack of future perspectives, and symptoms of depression [3, 31, 35]. Giannopoulou et al. [3] found that waiting for asylum in Europe through family reunification procedures and lower levels of social support predicted higher levels of depressive symptoms and negative cognitions among unaccompanied refugee minors. Similarly, Poole et al. [35] identified increased time in the asylum process in Greece as a significant risk factors for depressive symptoms among adult Syrian asylum seekers. In a recent study comparing peer-refugee helpers and non-helpers, Lavdas et al. [36] found similar levels of anxiety and depression across both groups, and that among refugee helpers, paid roles, a stronger sense of coherence, greater coping flexibility, and higher levels of social support were associated with fewer symptoms of anxiety and depression.

### Mental health and psychosocial well-being among asylum seekers and refugees in Greece

Psychological and social distress among asylum seekers and refugees can manifest in a wide range of emotional, cognitive, physical, and behavioural and social problems. Our review did not identify any systematic or large-scale epidemiological studies assessing the prevalence of mental disorders among asylum seekers and refugees in Greece. No longitudinal studies were found. The existing evidence base largely consists of small-scale cross-sectional studies, which vary considerably in terms of methodology, study setting, and target population group. Caution is therefore necessary when interpreting the available data.

Overall, the reviewed studies consistently reported a high burden of mental health concerns, including symptoms commonly associated with PTSD, depression, and anxiety. In addition, studies reported other serious mental health issues, including suicidal ideation, self-harming behaviours, substance use and psychotic symptoms. These findings are in line with trends observed in other refugee-hosting settings [7, 8].

#### Prevalence of mental health problems

##### Cross-sectional surveys

Available studies found high mental health burdens among asylum seekers and refugees in Greece. An overview of the available data from cross-sectional surveys with asylum seekers and refugees in Greece is presented in Table 2. Severe psychological distress was reported by more than 84% of participants in three large surveys [1, 37, 38], including a representative study of individuals registered with UNHCR across Greece [1]. Among studies investigating specific mental health conditions, rates of PTSD and depression have been the most frequently reported. In the available studies, rates for PTSD ranged from 35% to 81% [3, 33, 39, 40], with PTSD symptoms particularly prevalent in one study among unaccompanied refugee minors [19]. Reported rates for depression were similarly high and ranged from 33% to 67% in the available studies [3, 33, 35, 39]. Limited data are available regarding other mental health conditions. One study conducted in a refugee camp in a rural area in central Greece found rates of 27.9% for generalized anxiety disorder and 33.6% for insomnia [39].

**Table 2:** Available cross-sectional epidemiological surveys on mental health conditions among asylum seekers and refugees in Greece

Study	Sample	Setting	% above cut-off score (instrument)		
			Depression	PTSD	Psychological distress
Casalis et al., 2023	3755 individuals mainly from Afghanistan (27%), Syria (20%), and Iraq (9%) 42% female 39% between 19–29 years	Refugees located in various accommodations across Greece; sample representative of individuals registered with UNHCR database as of 2021			85% (Kessler-6)
Farhat et al., 2017	1293 individuals mainly from Syria (56.6%), Afghanistan (16.3%), and Iraq (15.7%) 40.1% female Median age: 19 years (640 refugees > 14 years old were screened for mental health)	Ritsona camp, Malakasa camp, Katsikas camp, Faneromani camp, Hotel Ioannina, Samos hotspot, Soho Hotel			84.9% (RHS-15)
Giannopoulou et al., 2022	90 unaccompanied refugee minors mainly from Syria (51.1%) 15.6% female Mean age: 16.2 years; Age range: 13–17 years	Nine long-stay residential facilities (shelters) for UMRs in Athens, run by a NGO	47.7% (DSRS)	81.1% (CRIES-8)	
Gordon et al., 2023	500 refugees mainly from Afghanistan (34.4%) and the DRC (20.8%) 35.0% female 59.2% between 18–30 years	Samos reception center			82.4% (Single question)
Knappe et al., 2023	150 refugees from Southwest Asia (68.0%) and Sub-Sahara Africa (32.0%) 50% female Mean age: 29.1 years	Refugee camp in rural area central Greece	33.3% (PHQ-9)	35.3% (IES-R)	
Maria et al., 2021	64 refugees mainly from Syria (66.4%) 37.5% female Mean age: 36 years	Apartments run by UNHCR in Larisa town	67.2% (PHQ-9)	54.7% (HTQ)	
Poole et al., 2018	135 refugees mainly from Syria (88.9%) 40.7% female Median age: 30	Camp for Syrian refugees located in the Attica region	43.7% (PHQ-8)		
Theofanidis et al., 2022	73 refugees from Syria 27.4% female 54.8% between 18–34 years	Refugee camp in northern Greece with a population of∼2,000 refugees		64.6% (PCL-C)	

Note. CRIES-8: Children’s Revised Impact of Event Scale; DSRS: Depression Self-Rating Scale; HTQ: Harvard Trauma Questionnaire; IES-R: Impact of Event Scale – Revised; Kessler-6: Kessler Psychological Distress Scale; PCL-C: PTSD Checklist-Civilian Version; PHQ-9 / PHQ-8: Patient Health Questionnaire; RHS-15: Refugee Health Screener-15

##### Analysis of case documentation in (mental) health services

Analysis of mental health complaints of asylum seekers and refugees presenting to health care services can potentially provide further important insights. Table 3 provides an overview of the available information. In line with findings from cross-sectional surveys of the general refugee population, symptoms of depression, anxiety, and PTSD were the most frequently identified symptoms among individuals presenting to services. Notably, a significant number of individuals presented to health services in acute mental health crises, characterized by suicidal and self-harm behaviours, severe panic and agitation, as well as psychotic symptoms [41–43]. In a study examining 218 mental health-related consultations at a clinic in Moria camp on Lesvos, 17% of consultations addressed acute mental health crises [43]. Available data from a mobile mental health service on Chios island suggests that one in three adult refugees referred to the service reported suicidal or self-harm behaviours [41]. Across studies, the reported rates of self-harm behaviour ranged from 7% to 13% [19, 42, 43], with the highest rates observed in a study among unaccompanied refugee minors [19]. A study conducted among 900 asylum seekers and refugees attending a NGO-provided mental health service on Lesvos found that one in three reported experiencing suicidal thoughts, and one in five had attempted suicide either before arriving in Greece, while residing in the camp, or both [42]. Findings by Hermans et al. from NGO-provided medical services on Lesvos suggest that the rate of hospital-treated suicide attempts among asylum seekers and refugees in Greece is higher than that reported among refugee populations in other European countries [44]. Psychotic symptoms were identified in 6% to 12% of asylum seekers and refugees presenting to health care services [19, 42]. Limited information is available on alcohol and substance use. Among asylum seekers and refugees referred to a mental health service, 2.6% of adults and 0.5% of children were diagnosed with mental and behavioural disorders due to psychoactive substance use [41].

**Table 3:** Available information on symptoms and diagnosis among asylum seekers and refugees presenting to health care services for mental health problems

Study	Sample	Setting	Type of analysis	Findings on mental health problems
International Rescue Committee, 2020	904 refugees consulting a mental health centre mainly from Afghanistan (35%), the DRC (13%) and Syria (8%) 40% female	Lesvos hotspot	Analysis of case documentation	67% sleeping problems 61% symptoms of depression 60% experiencing anxiety 41% symptoms of PTSD 35% suicidal thoughts 18% suicidal attempts 12% psychotic symptoms 9% self-harm
Giannopoulou et al., 2024	469 unaccompanied asylum-seeking children identified with mental health problems by field psychologists	Long-term accommodation in shelters and supported independent living apartments	Mental health needs as documented by field psychologists	18.6% Alcohol/Substance use 17.7% Depression symptoms 16.8% Violent behaviour 15.1% PTSD symptoms 13.0% Self-harm behaviour 9.6% Anxiety/Panic attacks 6.0% Psychotic symptoms 3.0% Suicide attempt
Van De Wiel et al., 2021	143 refugees identified with mental health problems at an emergency medical care clinic mainly from the DRC (44.7%), Iraq (21.3%), and Syria (16.0%) 37.3% female Mean age: 24.7 years	Moria camp	Analysis of type of mental health consultations	28.9% Trauma related symptoms 23.4% Mild to moderate agitation/dissociation/psychosis 11.9% Low mood and/or suicidal ideation 6.9% Self-harm 6.4% Severe panic/agitation/dissociation/psychosis 3.7% Suicide attempt
Fylla et al., 2022	418 refugees referred to a mobile mental health unit 55.5% adults mainly from Syria (23.3%), Iraq (21.6%), and African countries (17.2%) 35.8% female Mean age: 29 years 44.5% children mainly from Syria (37.1%) and Iraq (28.0%) 29.6% female Mean age: 12 years	Chios, Oinousses, and Psara	Analysis of diagnoses according to ICD-10 and suicidal behaviour as documented in case files	Adults: 2.6% Mental and behavioural disorders due to psychoactive substance use (F10–19) 3.0% Schizophrenia and related disorders (F20–29) 27.2% Mood(affective) disorders (F30–39) 42.7% Anxiety, dissociative, stress-related, somatoform, and other non-psychotic mental disorders (F40–48) 16.8% Others 7.8% Z-codes 34.0% Suicidal behaviour (defined as death wishes, self-injuries, suicide attempts) Children: 0.5% Mental and behavioural disorders due to psychoactive substance use (F10–19) 0.5% Schizophrenia and related disorders (F20–29) 10.8% Mood (affective) disorders (F30–39) 39.8% Anxiety, dissociative, stress-related, somatoform, and other non-psychotic mental disorders (F40–48) 25.3% Disorders of psychological development (F80–89) 15.6% Others 7.5% Z-Codes

### Coping and help-seeking patterns

Coping and help-seeking patterns among asylum seekers and refugees in Greece reflect a complex interplay of individual, social, cultural, and structural factors. While the refugee population in Greece is highly diverse, encompassing individuals from a wide range of regions and cultural backgrounds, it should be noted that the reviewed literature has focused predominantly on individuals from the Middle East. Therefore, the information presented here should be interpreted with caution and supplemented by more population-specific and in-depth evaluations in the future. In the available studies, addressing mental health concerns was often viewed by asylum seekers and refugees as a need for restoring safety, dignity, and a sense of belonging [2, 19, 32]. Accordingly, many individuals expressed a preference for non-clinical interventions, such as improved living conditions, access to basic services and healthcare, and community-based support systems, over formal psychological or medical treatment alone [32, 37].

#### Informal healing practices and resources in the communities

The reviewed literature highlights family and community networks as important sources of emotional support among asylum seekers and refugees in Greece. Social support was described as playing a crucial role in reducing social isolation, offering practical guidance, and fostering a sense of belonging [32, 37]. However, it is noted that the dynamics of displacement often challenge or disrupt these support systems by separating families and limiting access to trusted social networks [32]. Despite these challenges, solidarity often emerges among individuals with shared experience in the camps, creating informal support systems that help to cope with daily stressors [32, 37]. Women particularly emphasized the importance of peer-support groups and safe spaces for emotional expression and collective coping [32, 45]. Some individuals, however, hesitate to share their struggles with family or community members due to fear of stigma or burdening others [32]. For others, engaging in community networks was seen not only as a source of emotional coping but also as a way to regain a sense of self-efficacy and purpose in an otherwise disempowering environment [32, 45].

Religious practices were also described as a resource for coping. Faith in a higher power was described as a source of comfort and hope, helping individuals to make sense of their suffering and maintain a sense of control amid uncertainty [37]. Communal religious rituals, such as prayers, provided a sense of continuity, cultural identity, and belonging [37].

In addition to social and religious support, asylum seekers and refugees in Greece describe individual coping strategies to manage psychological distress [32, 37]. This includes cognitive reframing, such as focusing on future goals and regaining agency of their lives, as well as engaging in activities such as learning new skills or taking on helpful roles within the community [32, 37].

#### Utilization of MHPSS services

Despite high levels of psychological distress among asylum seekers and refugees in Greece, formal help-seeking is reported to be rather limited in the available literature [37, 41]. While some individuals view formal mental health services as an effective solution and actively engage with them when available, others point to the inconsistent availability of services and question their relevance in addressing the broader MHPSS-related problems they experience [31, 32].

In the reviewed literature, data on service utilization rates are limited, but available findings suggests that uptake remains low, even when mental health needs are identified [19, 37, 41]. A survey conducted across seven camps found that more than one third of individuals who screened positive for psychological distress refused referral to an on-site psychologist, and even fewer showed up for a consultation [37]. Similarly, data from a mobile mental health unit operating on Chios Island revealed significant dropout prior to first contact, with one-fifth of referrals cancelled before an appointment could be scheduled [41]. Notably, it is reported that individuals with heightened vulnerability, such as survivors of gender-based violence, torture, shipwrecks, or those who lost relatives at sea, as well as those with acute mental health crises were more likely to seek formal support [42, 44].

### Barriers to accessing MHPSS services and quality of care

Table 4 describes common barriers to accessing MHPSS services and achieving quality care identified in the review process. The identified barriers have been reported by both service providers and humanitarian stakeholders [19, 20, 41, 42, 46], as well as asylum seekers and refugees [31, 32, 37]. The identified supply-side barriers are complex and stem from both general constraints of the health system and a lack of responsiveness to the specific needs of refugee and asylum-seeking populations [20, 24, 28, 47]. In addition, demand-side barriers such as perceptions of mental health care and stigma within refugee communities can further hinder access and engagement [31, 32, 37]. Importantly, many of these barriers are interconnected and mutually reinforcing. For example, barriers within the health system, such as the absence of trained interpreters or culturally sensitive services, can lead to perceptions among asylum seekers and refugees of MHPSS as inadequate or irrelevant to their needs [31, 32, 37]. The same applies when MHPSS services are perceived as using medicalized concepts of mental health and focusing solely on symptoms of distress, rather than addressing the broader context, such as poor living conditions or ongoing uncertainty [31, 32, 37]. This, in turn, can reduce the likelihood that services will be viewed as trustworthy or helpful, even when they are technically available.

**Table 4:** Common barriers to accessing MHPSS services and quality of care for asylum seekers and refugees in Greece

Supply-side	Demand-side
**Approachability** *“Do services enable people in need to identify that offers for care exist*,* can be reached*,* and can improve health?”*	**Ability to perceive** *“Do people recognize their need for care?”*
• Lack of provision of information about available services • Complex mix of camp-based services, public services, private services and NGO services	• Knowledge about mental health • Diverse cultural beliefs and interpretation of mental health problems • Perceptions of severity of symptoms
**Acceptability** *“Are services structured and organized in such a way that those seeking care are inclined to use them and perceive them as suitable for their needs?”*	**Ability to seek** *“Do individuals’ express their intention to obtain health care?”*
• Often represent medicalized concepts of mental health • Lack of service providers that share the same cultural background or lived-experience • Lack of interpreters	• Stigma around mental health • Fear of stigma around mental health seeking, MHPSS services are seen as associated with severe mental illness • Preference for community support • Distress of living conditions making it difficult to find strength and motivation to seek help from mental health service • Professionals are perceived as unfamiliar with cultural background and experiences • Unfamiliarity with health care system and available services • Difficulties navigating services • Perception that service use could negatively influence asylum procedures • Lack of knowledge about services and potential benefits
**Availability, accommodation and affordability** *“Can services be reached both physically and in a timely manner? Do the services have the necessary capacity and resources to provide care?”*	**Ability to reach and pay** *“Can people physically reach service providers? Can people generate economic resources to pay for health services”*
• Severe staff shortages in camps and public mental health services • Lack of specialists (e.g. psychiatrists) • Delays in PAAYPA / AMKA issuance • Capacity constraints in mental health services in camps and outside, especially on islands • No structural integration or coordination between camp services, public services, and NGOs • Camps are in remote locations with large distance to healthcare facilities • Long waiting times to see professionals	• Public transport not available • Difficulties to navigate transport systems due to language barriers
**Appropriateness** *“Is service quality assured and is there a fit between services and clients’ needs?”*	**Ability to engage** *“* *Do patients participate in decision-making and treatment decisions?”*
• Language barriers • Lack of interpreters • High mental load and burnout among staff • Short consultations, limited follow-up • Staff understanding of MHPSS needs among asylum seekers and refugees • Staff understanding of specific experiences of refugee and asylum-seeking women • Lack of continuity of care through lack of coordination between services • Racist/discriminatory behaviour in services	• Perception that services don’t address structural needs • Low trust due to past negative experiences • Services perceived as ineffective or irrelevant in addressing difficulties • Often high mental health needs, that require long-term care in a safe environment • Scepticism toward mental health professionals unfamiliar with their background

## Discussion

This review synthesised available evidence on MHPSS for asylum seekers and refugees in Greece. It makes a distinctive contribution by bringing together dispersed evidence on MHPSS for asylum seekers and refugees in Greece, a critical context within the European migration landscape, and by integrating findings on mental health needs, service availability, and barriers to care. Our findings highlight the influence of social determinants on mental health outcomes, the high prevalence of psychological distress, and gaps in access to appropriate services. These insights can guide the planning, coordination, and delivery of more effective and equitable MHPSS for asylum seekers and refugees.

Asylum seekers and refugees in Greece experience high levels of psychological distress. Although no large-scale epidemiological studies on mental disorder prevalence among refugees in Greece were identified, available evidence aligns with systematic reviews across refugee populations in other hosting contexts [7, 8]. Reported rates of mental health problems among refugees in Greece are substantially higher than those observed in the general population of Greece [48]. The elevated rates of mental health problems among refugees in Greece appear to be driven by cumulative exposure to stressors throughout the migratory journey. Consistent with global evidence [49, 50], the combination of pre- and peri-migration trauma and ongoing post-migration stressors contributes to heightened vulnerability. Post-migration and chronic daily stressors - such as insecure legal status, inadequate housing, and barriers to social inclusion - negatively affect mental health, compound the impact of pre-migration trauma, and can hinder recovery [49, 50]. These findings suggest that a predominantly medicalized framing of refugee mental health risks neglecting broader social, legal, and political determinants of mental distress. Our findings therefore underscore the critical role of structural and social determinants in shaping mental health outcomes for asylum seekers and refugees in Greece and, in line with the IASC MHPSS framework, point to the need for MHPSS approaches that complement individual clinical care with rights-based and strengths-oriented strategies promoting dignity, participation, and social connectedness [51].

The reviewed literature consistently indicates that MHPSS service provision for asylum seekers and refugees in Greece remains limited, with substantial gaps in access and continuity of care. Application of Levesque’s patient-centered access framework [17] revealed barriers across key access dimensions such as approachability, availability, and appropriateness. The barriers identified, including limited healthcare entitlement, remotely located accommodation of refugees and discriminatory practices within the health system, reflect the structural determinants of health that shape inequitable distribution of resources. While Greece faces unique challenges as a primary entry point, similar inequities exist across Europe. For example, our findings align with patterns in other European host countries, where MHPSS for refugees is often excluded from national health systems and constrained by restrictive migration policies [9, 10]. Such systemic barriers, including those observed in the present review, reinforce mental health inequities and, if unaddressed, will not only exacerbate individual psychological distress but also impose an increasing long-term burden on public health systems.

Community-based support plays an important role for asylum seekers and refugees in Greece. Across the reviewed studies, asylum seekers and refugees frequently relied on family and community networks, religious practices, and informal support systems. Systematic reviews similarly highlight the importance of family and community support for mitigating stressors and promoting mental health in displacement settings [52, 53]. Our findings underscore the need to scale up MHPSS approaches that are culturally responsive, community-driven, and sensitive to the displacement context. Recent research suggests that training peer support workers and implementing scalable interventions can be both effective and contextually appropriate for addressing mental health needs among refugees in Greece [45, 54].

A key challenge to delivering adequate care within formal MHPSS services identified in this review relates to workforce capacity. Shortages of trained providers, limited cultural competence and insufficient training in trauma-informed approaches constrain the provision of equitable and effective care [19, 31]. These findings are consistent with previous evidence on the preparedness of mental health professionals to meet the needs of migrant and refugee populations [9, 55]. Stress and burnout among healthcare workers working with asylum seekers and refugees in Greece can further compromise service provision [56]. Strengthening workforce capacity and improving working conditions are therefore critical steps toward reducing inequities in access and quality of MHPSS for refugees.

While several studies on the key topics of this review were identified, there remains a lack of large-scale, population-specific, and longitudinal data on MHPSS in Greece. Similar evidence gaps are documented across other refugee-hosting countries [9, 10, 57], limiting the ability to identify inequities and improve support systems. Without comprehensive and disaggregated data, many inequities remain invisible, constraining evidence-informed policymaking and system strengthening. Strengthening data collection, monitoring, and evaluation mechanisms is therefore essential to advance mental health equity for asylum seekers and refugees.

### Implications for policy and practice

#### Recognizing and addressing the social determinants of mental health

- Equitable access to essential services (such as safe shelter, food, health care, water and sanitation, legal protection, education, and livelihood opportunities) is required as meeting basic needs is foundational to promoting mental health and psychosocial well-being [16].
- Promote social inclusion through policies and programs that strengthen community networks, foster peer support, and build connections between refugee and host communities to reduce discrimination and social tensions.
- Integrate mental health considerations into core policies, strategies, and programmes across sectors, in line with WHO guidance on cross-sectoral promotion of mental health [58].

#### Strengthening mental health service systems

- Establish monitoring mechanisms and regular data collection on service provision, staffing, and gaps, with attention to underserved areas such as camps and remote settings.
- Integrate MHPSS for asylum seekers and refugees into national health and social service systems, ensuring enabling resources such as interpreters and cultural mediators.
- Develop standards for referral pathways and coordination between camp-based services, NGOs, and public health institutions to improve continuity of care.
- Platforms for regular exchange between health, protection, education, social services, and legal sectors can facilitate intersectoral collaboration.

#### Improving mental health service delivery

- Strengthen existing community-based resources by adopting community-support models and rights-based approaches, as recommended by WHO [59].
- Aligning interventions with the beliefs, practices, and needs of the refugee and migrant populations can help to ensure that MHPSS services are culturally relevant and context sensitive.
- Expand the use of evidence-based, scalable psychological interventions. Interventions developed by WHO, such as Problem Management Plus (PM+), Early Adolescent Skills for Emotions (EASE), Doing What Matters in Times of Stress (DWM), and Self-Help Plus (SH+), can be considered for broader implementation in both camp and community settings. Guidance is available on how to integrate these interventions into existing services [60] and several interventions have already been translated to Greek and culturally adapted for refugees in Greece [61]. Existing MHPSS coordination platforms may provide important entry points for contextual adaptation and coordinated implementation across actors.
- Define and implement quality standards for services to prioritise areas for quality improvement, including co-design approaches that meaningfully engage people with lived experience of forced displacement - such as through advisory groups, peer roles, or participatory feedback mechanisms - in the development of culturally appropriate mental health services [62].

#### Strengthening the mental health workforce

- Comprehensive training programs should equip specialized MHPSS providers with the skills and knowledge needed to deliver high-quality, culturally sensitive, and trauma-informed care.
- Build capacity among general health care providers and non-specialised providers, including peer refugee helpers, using tools such as the mhGAP Humanitarian Intervention Guide [63] and the WHO-UNICEF EQUIP platform [64].
- Implement ongoing supervision and support structures for MHPSS providers, including peer refugee helpers, alongside strategies to promote provider well-being and prevent burnout.

#### Advancing data on mental health and psychosocial support

- Expand research on MHPSS needs, barriers, and service delivery for asylum seekers and refugees in Greece.
- Prioritize quantitative and population-specific studies, ensuring that research captures the experiences of diverse refugee groups across different camp and community settings [57].
- Complement quantitative evidence with qualitative and participatory research approaches to capture lived experiences and service user perspectives, thereby supporting culturally responsive MHPSS interventions.
- In-depth research focused on vulnerable groups, such as unaccompanied minors, survivors of gender-based violence, and individuals with disabilities can help to better tailor services to specific needs.

### Limitations

Several limitations must be acknowledged. First, the reception conditions and asylum and migration policies have been rapidly changing in Greece in recent years. As a result, some of the information reviewed may already be partially outdated. Second, this review followed a narrative approach to synthesis evidence across different key topics relevant to MHPSS. While the methodological approach provided rich information, this review should not be regarded as systematic review of all issues related to MHPSS of asylum seekers and refugees in Greece. The possibility that additional valuable information may not have been identified or included in this review cannot be ruled out. It is also important to emphasize that the refugee and asylum-seeking population in Greece is highly heterogeneous, encompassing individuals from a wide range of countries of origin, cultural backgrounds, and migration experiences. As such, the findings presented in this review may not be equally applicable to all subgroups within this population. The information should be interpreted in conjunction with population-specific MHPSS assessments, such as those conducted for Syrians affected by armed conflict [65]. Finally, we did not conduct a quality appraisal of the included studies. The evidence base reviewed largely relies on qualitative and small-scale cross-sectional studies, with limited longitudinal or large-scale epidemiological research available.

## Conclusions

This narrative review provides the first comprehensive synthesis of evidence on MHPSS for asylum seekers and refugees in Greece, addressing major stressors, needs, services, and barriers to care. High levels of mental health needs were identified, associated with pre-migration experiences and exacerbated by the daily stressors of resettlement in Greece. There is an urgent need to address these challenges to support the full participation and meaningful integration of refugees and asylum seekers into society. Our review situates Greece’s context within broader European trends, underscoring common gaps and opportunities for integrated, culturally responsive care. The proposed actions outlined in this review may serve to inform policymakers and practitioners seeking to strengthen MHPSS systems and reduce mental health inequities among displaced populations.

## Data Availability

Data sharing is not applicable to this article as no datasets were generated or analysed during the current study.
